# Biomechanical Evaluation of Wasp and Honeybee Stingers

**DOI:** 10.1038/s41598-018-33386-y

**Published:** 2018-10-08

**Authors:** Rakesh Das, Ram Naresh Yadav, Praveer Sihota, Piyush Uniyal, Navin Kumar, Bharat Bhushan

**Affiliations:** 1 Department of Mechanical Engineering, Indian Institute of Technology (IIT) Ropar, Nangal Road, Rupnagar, Punjab 140001 India; 2 Nanoprobe Laboratory for Bio- & Nanotechnology and Biomimetics (NLBB), The Ohio State University, 201 W. 19th Avenue, Columbus, OH 43210-1142 USA

## Abstract

In order to design a painless and mechanically durable micro syringe-needle system for biomedical applications, the study of insect stingers is of interest because of their elegant structures and functionalities. In the present work, the structure, mechanical properties and the mechanical behavior during insertion of wasp and honeybee stingers have been investigated. The non-invasive imaging tool, micro-computed tomography has been employed to reveal the 3D-structures of wasp and honeybee stingers. A quasi-static nanoindentation instrument was used to measure the nanomechanical properties. Both wasp and honeybee stingers have graded mechanical properties, decreasing along their longitudinal direction starting from the base. The computed tomography images and the measured material properties from nanoindentation were fed into a computational framework to determine the mechanical behavior of the stingers during penetration. The computation results predicted the penetration angle of +10° for the wasp stinger and −6° for the honeybee stinger, which mimics the practical insertion mechanism of both stingers. Based on this understanding, a wasp and honeybee stringer inspired micro syringe-needle design has also been proposed.

## Introduction

Some of the arthropods sting into prey and release a venom in them and are classified as stingers. Some of the common arthropods which use stingers are honeybees, wasps, and scorpions^1,2^. Honeybees and wasps sting using a pair of stylets, which move back and forth alternatively while stinging into its prey. The stylet is a medical name for the slender, piercing part. To release the venom, both use the opening formed between the stylets. Honeybees and wasps sting and release venom in a similar fashion^1^. Scorpions use a sharp tail (aculeus) to sting into their prey^2^. The tail has an opening which releases venom to paralyze the prey. The chemical composition, geometrical morphologies and biological functions of paper wasp and honeybee stingers have been reported in literature^1^. During years of evolution, these stingers have adapted for different mechanical and biological functions e.g. attack, prey carriage, defense, mating, cutting, etc.^1^. These stingers have similarity in their composition and biological activities but their structures and insertion skills are measurably different. It has been reported that the proximal force required for a honeybee stinger or a wasp stinger to pierce the skin is on the order of 2–3 mN and 6–8 mN, respectively^1^. The stingers have tapered geometry, shape and refined insertion mechanics, which allow them to penetrate with very low puncturing force preventing the mechanical failure^1^.

The focus so far has been on the chemical composition, geometrical morphology and biological functions of wasp and honeybee stingers^1,3^. The mechanical properties (such as variation of modulus and hardness along the length and cross-section), 3D structure along the transverse and cross section directions, sliding and guiding channels, venom micro channels of wasp and honeybee stingers still are not known. The mechanics behind the specific penetration angle of these stingers is also not known. All these are significant for bioinspired design of needles, and also of interest in evolutionary biology. In addition, no relevant literature on modelling based on the computed tomography images of honeybee and wasp stingers exist.

In the present study, static nanomechanical properties mapping of the wasp and honeybee stingers, structure, and the mechanical behavior during penetration have been studied. The nanomechanical characterization of wasp and honeybee stingers has been performed employing quasi-static nanoindentation instrument. The average elastic modulus and hardness and their mapping on the surface along the longitudinal and cross sectional directions of the stingers have been obtained. The structures of these stingers have been analyzed using a 3D-micro computed tomography technique. In addition, the mechanical deformation and stress distribution in the stingers, under compressive loading conditions, have been modelled by feeding its 3D-computed tomography images. A comparison of these properties between wasp and honeybee stingers is presented. Based on the understanding of these investigations, a design of a micro syringe-needle is presented. The structures, nanomechanical properties, and mechanical behavior during penetration of stingers will inspire the product designers to fabricate various biomedical devices like micro syringe-needle system for drug delivery, hard tissue biopsy needle, surgical suture etc., with reduced penetration force to prevent tissue damage.

## Experimental

### Sample preparation

The wasps (*Vespula vulgaris*) and honeybees (*Apis cerana*) were collected from the nearby region of the Indian Institute of Technology Ropar, Punjab, India (30.9664°N 76.5331°E). These samples were preserved in a deep freezer in a 50% ethanol solution between the time of collection and analysis. The stingers were extracted from the female wasps and worker honeybees, located on the posterior end. The stingers were cleaned properly and dehydrated (kept in a sealed tube at room temperature (~25 °C) for an hour and the solvent was allowed to evaporate) before experimentation. The specimen preparation procedure has been followed from previous research^4,5^, and this does not affect the physical properties of the stingers.

### Structure analysis through 3D micro-computed tomography (µ-CT)

The 3D micro-computed tomography (µ-CT) imaging of the stingers was performed using GE phoenix nanotom s µ-CT machine. The wasp stinger samples were scanned at a resolution of 10 µm at 280 µA and 29 kV, and the honeybee stinger samples were scanned at a resolution of 2.5 µm at 410 µA and 30 kV. The 3D scanned images were segmented based on grey value in Simpleware Scan IP software to a build a 3-D model. In this segmentation process, the built-in median filter and the flood fill tool were used to reduce noise.

### Nanomechanical property measurements of stingers

The nanoindentation instrument was employed for quasi-static nanomechanical property measurements (elastic modulus and hardness) of stingers^6,7^. A total of five (5) samples of both wasp and honeybee stingers were measured. The stinger has intrinsic curvature and to avoid this curvature effect, the entire stinger was cut into three sections (basal, medial and apical regions) and the ventral surfaces of each were put on the semi-solid epoxy block and stuck on it carefully. After 2 hours, the epoxy was cured and the samples were embedded in it. The embedded samples were examined by an optical microscope to ensure that they were fixed properly. Then, on the flat region of dorsal surface, an attached scanning probe microscopic (SPM) was utilized to scan 15 µm area for each indenting location and the indentation experiment was performed using TI 950 Tribo-Indenter (Hysitron, Inc., Minneapolis, MN, USA) with standard Berkovich diamond tip (radius of curvature ≈ 150 nm). The calibration of the instrument was performed with the help of standard fused quartz and polycarbonate samples following the standard procedure^6^.

To obtain nanomechanical property maps of the wasp stingers, a total of sixty (60) indentations were performed along the longitudinal direction of the stinger, starting from base (basal region) towards tip (apical region) with an indent spacing of 40 µm. Indentation measurements were made on three (3) parallel longitudinal lines with spacing of 4 µm. Thus, the elastic modulus and hardness were measured at 3 × 60 points and matrices of elastic modulus and hardness were formed in the order of 3 × 60. The contour plots of the matrices were generated using MATLAB to get the 2-D mapping of elastic modulus and hardness of the stinger. The mapping images were superimposed onto the CT image of the stinger for more clear visualization of the modulus and the hardness variations along the longitudinal direction of the stinger.

In the case of the honeybee stinger, a total of forty (40) indentations were performed along the length, from base to tip, with a spacing of 30 µm. Indentation measurements were made along the length, on three (3) parallel lines with a spacing of 4 µm, which results in 3 × 40 measurement points on the surface. The same technique which was used for wasp stinger was repeated to get the mapping of the elastic modulus and hardness.

To perform the quasi-static nanoindentation at the cross section of the stingers, the stingers were vertically embedded within a small semisolid epoxy block where the tip was placed inside the epoxy and the base was kept just outside the top surface of the epoxy block. Samples of both the wasp and honeybee stingers were prepared in that way and their cross sections were observed using an SPM attached with the nanoindentation instrument. Only the samples in which the cross sectional surfaces became prominently focused were selected for nanoindentation from exocuticle to endocuticle with indent spacing of 2 µm. The elastic modulus and hardness mapping were also generated in this case.

The peak load for all these indentations was 10  µN. A load function consisting of a 5 sec loading to peak force segment, followed by a 2 sec hold segment and a 5 sec unloading segment was used. The load verses penetration curves were obtained from these experiments. The reduced elastic modulus or effective elastic modulus (E) and the hardness (H) were obtained from these curves.

### Mechanical analysis of stingers through numerical modelling

A numerical model was developed to study the effect of penetration angle on the deformation using measured mechanical properties. The segmented 3-D model of stingers was meshed in an FE model of Simpleware Scan IP advanced meshing software with tetrahedral elements (C3D4). The number of elements in this model was 332,453. This meshed model was exported to ABAQUS-FEA (version 6.10) package for finite element simulation. For simulation, the average material properties obtained from nanoindentation were used. During simulation, the stingers were considered as linear elastic and isotropic and were clamped at the base (all the rotational and translational degree of freedom constrained). The in-plane concentrated force of different amplitudes (10 mN, 30 mN, 50 mN, 80 mN and 100 mN) was applied along the central axis of the stinger at its tip and the resulting stress distribution, strain distribution and displacement, structural deformation/deformed shape were analyzed. The penetration angle of the applied force was varied from −90° to +90° and the corresponding stress and strain distribution were obtained.

In the second case, the gradient mechanical properties were applied to the stinger. In Scan IP, the 3-D model of the stinger was divided into three sections and every section was defined by individual masks, followed by meshing with C3D4 elements. After exporting the model to Abaqus, the three different nanoindentation moduli were applied to these three sections. Then the simulation was performed applying the same procedures as mentioned before.

## Results and Discussion

### Structural analysis of stingers by 3D micro-computed tomography (µ-CT)

**Figure 1:**
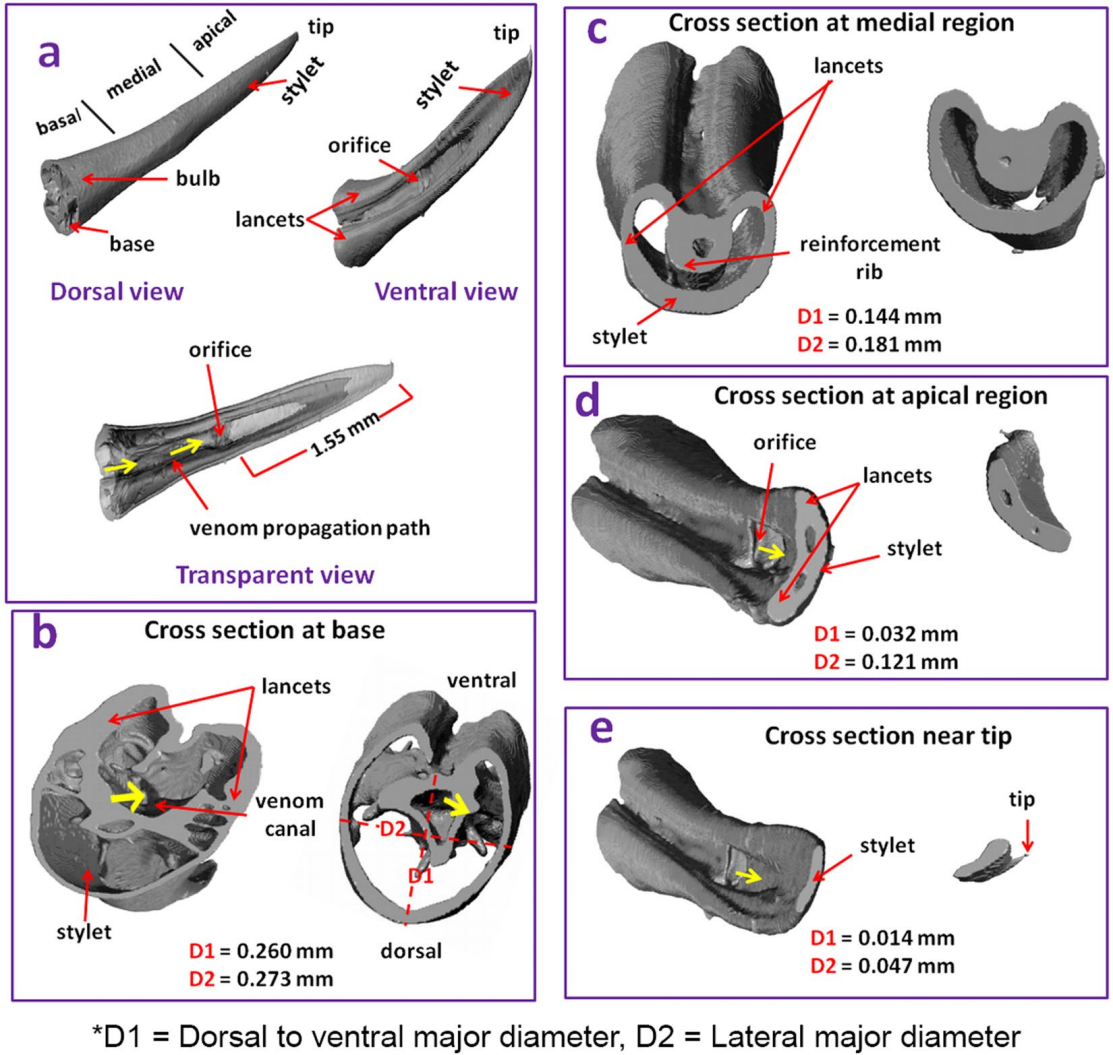
(**a)** Structure of whole wasp stinger obtained from micro-CT (**b**) Cross section at the base (**c**) Cross section at the medial region (**d**) Cross section at the apical region, and (**e**) Cross section near the tip. It is observed that the stylet and lancets have hollow shaft and their radii decrease from the base to tip. The hollow structures diminish near the apical region and are transformed into solid structures. The cylindrical hollow venom propagation path is also visible.

The µ-CT images of the different sections of the wasp stinger are presented in Fig. 1. Different views (dorsal, ventral and transparent view) of the wasp stinger are presented in Fig. 1a. The wasp stinger can be sectioned longitudinally into three regions; basal, medial and apical. The stingers have three major components; stylet, lancet and tip. There are paired lancets, one stylet and a tip. The wasp stinger has an intrinsic curvature with a bulb type base, a slender medial region having constant diameter, and an apical region with decreasing diameter and tapered tip. In the ventral view of the wasp stinger, the location of the natural orifice is pointed out, and in the transparent view, the venom propagation cylindrical path is designated by the yellow arrow. The venom is stored in the venom sack located at the lower abdomen of the wasp which is connected to the bulb of the stinger. The venom is transported through the hollow venom canal and transferred into the victim’s body through the orifice.

The wasp stinger has an average length of 2.67 ± 0.38 mm. The dorsal to ventral major diameter is denoted by D1 and the lateral major diameter is denoted by D2. The values of D1 and D2 in all regions are presented in Fig. 1. The lateral diameter (D2) of the wasp stinger in all regions is greater than the dorsal to ventral diameter (D1). The diameters determined here are the average diameters of all five (5) wasp stinger samples. The wasp pierces approximately 1.55 mm length section of its stylet into the victim’s skin as shown in the transparent view of the stinger in Fig. 1a. This length has been referred based on the height of the venom orifice from the tip of the stinger. It shows that the two lancets converge in the apical region. The cross section of the base of the wasp stinger is shown in Fig. 1b. Additionally, the cross sectional view of the basal region from the inside of the wasp stinger has been obtained through the post processing of the µ-CT image by Scan IP. Both of the cross sectional views of the base reveal the structure of the stylet, lancets and the venom canal. The stylet and lancets have hollow shafts and their radius decreases from the base towards the tip. These structural features make the stinger lightweight and strong. It has been reported in wasp and honeybee stingers, the hollow shaft makes them lightweight^1^. The gradient geometry of the sting like structures like scorpion stinger^2^ and the spider fang^8^ potentially improve their mechanical stability, durability and biological functions. Based on this, it is expected that the gradient geometry (decreasing cross section from base to tip) of the wasp and honeybee stinger, also improves mechanical stability and durability. The internal cross section at the medial region are shown in Fig. 1c. The internal cross sections are shown from two different directions, from inside to base and from inside to tip. A reinforcement rib is present in the ventral side of the medial region. This reinforcement improves the buckling resistance of the wasp stinger during its penetration into the victim’s body^1^.

**Figure 2:**
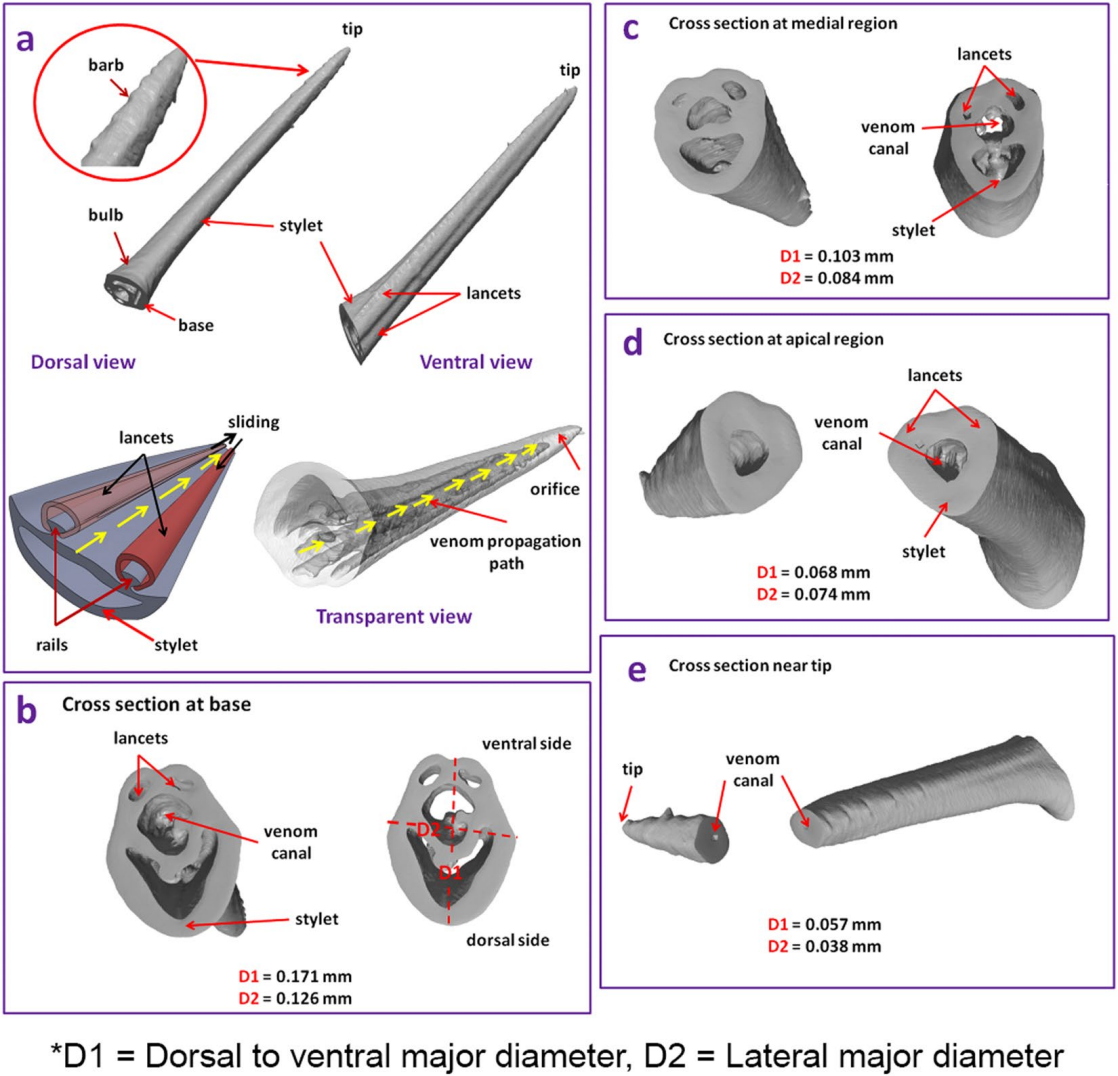
(**a**) Structure of the whole honeybee stinger obtained from micro-CT, (**b**) Cross section at the base, (**c**) Cross section at the medial region, (**d**) Cross section at the apical region, and (**e**) Cross section near the tip. The lancets freely slide onto the rails of the stylet as per schematic representation in (**a**). The stinger penetrates into the victim’s body because of the cooperative alternative motion of the paired lancets and the venom is pumped through the canal into the wound.

The µ-CT images of the different sections of the honeybee stinger are shown in Fig. 2. The dorsal, ventral and transparent views of the honeybee stinger are exhibited in Fig. 2a. Like the wasp stinger, the honeybee stinger is also sectioned into three regions (basal, medial and apical) and has three major components (paired lancets, one stylet and tip). The honeybee stinger has a straight geometry unlike the curved geometry of the wasp stinger and has the basal region, medial region, apical region and tip geometries similar to that of the wasp stinger. The length of the honeybee stinger is in the order of 1.62 ± 0.18 mm. The values of D1 and D2 in all regions are affixed in Fig. 2. In the honeybee stinger unlike the wasp stinger, the lateral diameter (D2) in all regions (except apical region) is less than the dorsal to ventral diameter (D1). The reverse facing barbs are distinctive and symmetrically distributed near the subapical region of the stingers as magnified and encircled in Fig. 2a. The reverse facing barbs are visible but are not prominent. As cited in a previous paper^3^, a row of barbs make an angle of 9° with the axis of the stinger shaft. The unique orientation of these barbs facilitates the penetration of the honeybee stinger into the objects. The barbs stick into the victim’s skin after insertion.

In Fig. 2a, the arrow designates the venom propagation path. As in the wasp, the venom of the honeybee is also stored in the bulb like venom sack and is transported through the hollow venom canal into the victim’s body. The stinger penetrates into the victim’s body due to cooperative alternative motion of the paired lancets and the venom is pumped through the canal into the wound^1,9^. The pumping and insertion mechanism are caused by the movements of the three paired plates which are controlled by the muscles. The moving plates drive the lancets to freely slide onto the rails of the stylet. The stylet has paired rails in which the grooves of the lancets are suitably fitted. The venom is discharged through the gap generated near the tip due to relative sliding motion of the lancets. The cross sectional views of the base from the outside and from the inner side are presented in the Fig. 2b. The lancets, stylet and venom canal all have hollow structures. The cross sectional views of the honeybee stingers at medial, apical and subapical regions (Fig. 2c–e) were obtained after post processing of the µ-CT images by the Scan IP software. However, as shown in Fig. 2d, at apical region, the hollow structure of the lancet pair and stylet diminishes and transforms into solid structure. The diameter of the venom canal gradually decreases after the apical region and becomes smaller near the tip.

To summarize, the 3D-structures, geometry with approximate dimensions, functioning mechanism of both wasp and honeybee stingers have been obtained from the µ-CT analysis. Both of the insect’s stingers have hollow structural components with gradient geometry which makes them elegant and mechanically durable. The wasp stinger has an intrinsic curvature and reinforcement rib at its medial region whereas the honeybee stinger does not have these features. Measured 3D structures of both wasp and honeybee stingers were directly fed into the computational framework for further biomechanical studies.

### Quasi-static nanomechanical analysis of the stingers

**Figure 3:**
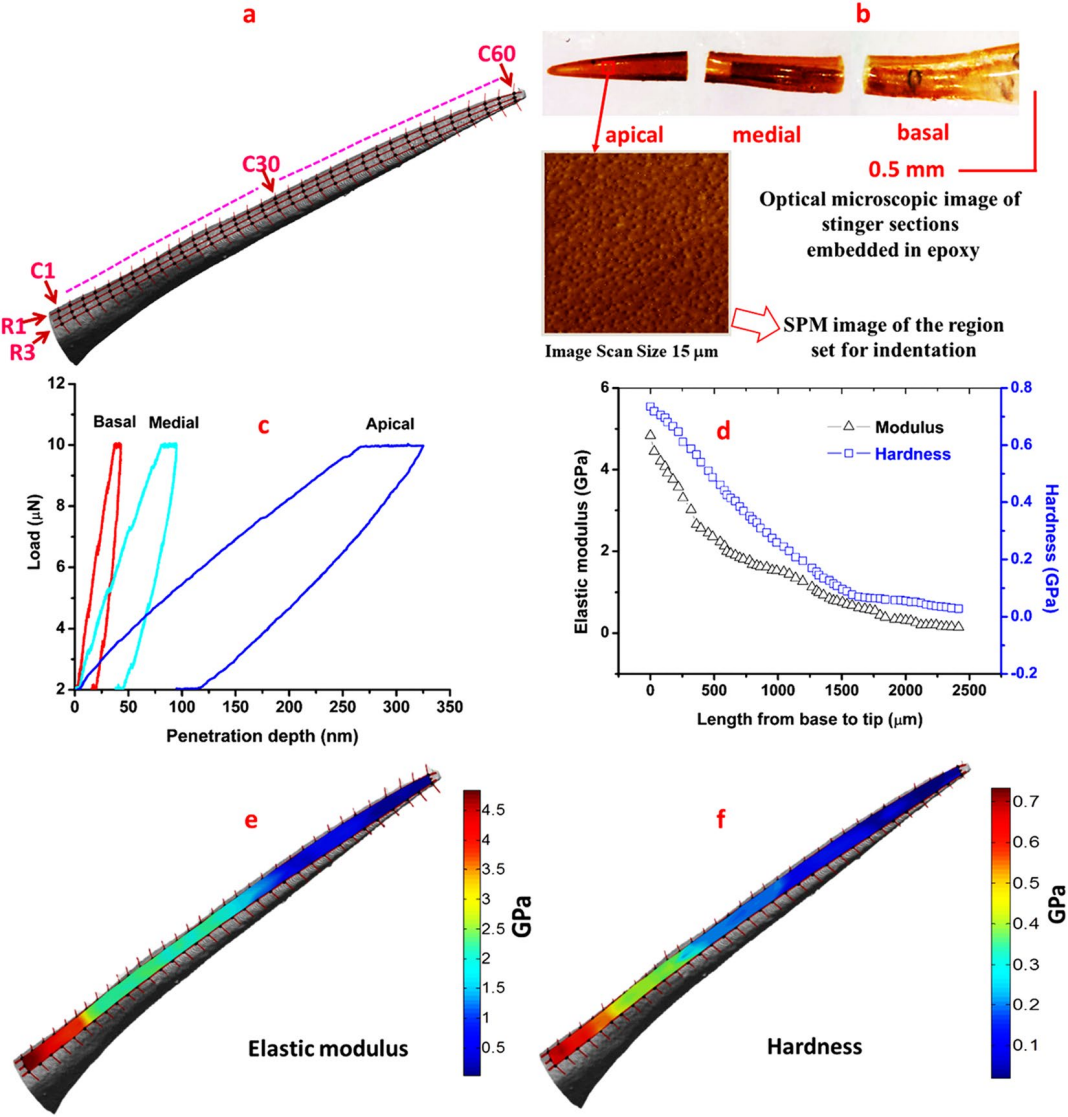
Quasistatic nanoindentation results of wasp stinger (**a**) Approximate sites of nanoindentation on stinger dorsal surface, (**b**) The optical microscopy images of the stinger sections embedded in epoxy and the SPM scan image of apical region used for nanoindentation (**c**) Load versus penetration depth curve at different regions of the stinger, (**d**) Variation of the Modulus and Hardness along the length from base to tip of the stinger, (**e**) Modulus mapping along the length, (**f**) Hardness mapping along the length. [The mapping clearly indicates the mechanical properties reduce along the length from base to tip of the stinger].

**Figure 4:**
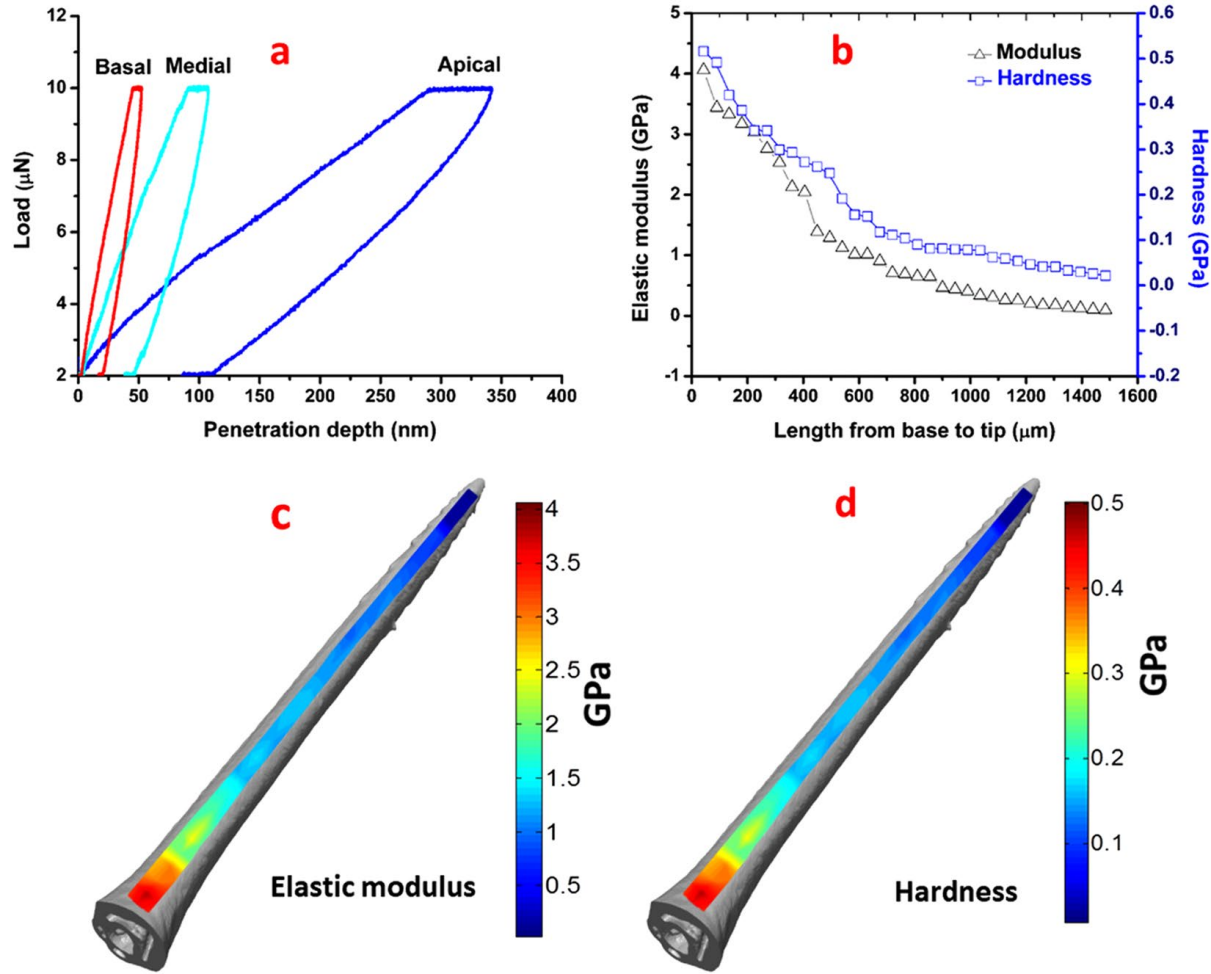
Quasi-static nanoindentation results of the honeybee stinger, (**a)** Load versus penetration depth curve at different regions of the stinger, (**b**) Variation of the Modulus and Hardness along the length from base to tip of the stinger, (**c**) Modulus mapping along the length, and (**d**) Hardness mapping along the length. The honeybee stinger has the same trend of mechanical properties along the longitudinal direction from base to tip as in the case of wasp stinger.

The quasi-static nanoindentation results of the wasp and honeybee stinger are shown in Figs 3 and 4, respectively. The approximate sites of nanoindentation are denoted by the nodes of the mesh depicted on the dorsal surface of the wasp stinger as shown in Fig. 3a. The optical microscopy images of the samples embedded in the epoxy and the SPM scan image of apical region used for nanoindentation are shown in Fig. 3b.

In Fig. 3c, the load versus penetration depth curves at the different regions (apical, basal and medial) on the dorsal surface of the wasp stinger are presented. These are the representative curves of three indentations each at basal, medial and apical regions. The penetration depth at the basal region is minimum. It increases along the length towards the tip and, at the apical region, it becomes maximum. In Fig. 4a, the load versus penetration depth curve of honeybee stinger is presented. For both wasp and honeybee stingers, the penetration depth at the basal region is minimum and is maximum at the apical region.

The average elastic modulus and hardness determined from the load versus penetration depth curves at three regions are shown in Table 1. The variation of elastic modulus and hardness of the wasp stinger, as a function of the length from the base to tip, is shown in Fig. 3d. The elastic modulus and hardness at the basal region of the stinger is maximum and both decrease along the length from base to tip, at the region near the tip it becomes minimum. Subsequently, the modulus mapping and hardness mapping of the wasp stingers are shown in Fig. 3e,f. The images of the elastic modulus and hardness mapping are placed on the µ-CT image of the wasp stinger. The mappings clearly exhibit the presence of the downtrend of elastic modulus and hardness along the longitudinal direction from base to tip of the stinger. Along the transverse direction, no measurable gradient of elastic modulus and hardness is observed.

**Table 1:** A summary of quasi-static nanoindentation results of wasp and honeybee stingers.

Region of interest	Wasp stinger	Honeybee stinger	Elastic modulus (GPa)	Hardness (GPa)
	Elastic modulus (GPa)	Hardness (GPa)		
Basal region	3.45 ± 1.36	0.56 ± 0.15	3.08 ± 0.89	0.42 ± 0.08
Median region	1.32 ± 0.44	0.31 ± 0.14	1.42 ± 0.62	0.22 ± 0.08
Apical region	0.47 ± 0.33	0.09 ± 0.03	0.42 ± 0.33	0.07 ± 0.05

In the case of honeybee stinger, as shown in Fig. 4b, both elastic modulus and hardness have negative gradients along the longitudinal direction from base to tip and there is no distinguishable gradient along the transverse direction as observed in the wasp stinger. The elastic modulus and hardness mapping are exhibited in the Fig. 4c,d. In the case of wasp and honeybee stingers, no such study on the nanomechanical analysis has been previously reported.

Like a scorpion stinger, it is predicted that during penetration both the wasp and honeybee stingers undergo compression, complex bending and twisting deformation, specifically the basal region is subjected to the largest values of bending stresses and axial buckling, so this region has the highest elastic modulus and hardness^2^. As per the structural analysis, the medial region of the wasp stinger has the intrinsic curvature along with a reinforcement rib which minimizes the bending moments and stresses^1^. In the case of the honeybee stinger, there is no such feature found at the medial region, thus the medial region of the honeybee stinger has a higher modulus than the wasp stinger. The wasp and honeybee stingers have the highest elastic modulus at their basal regions which decreases along the longitudinal direction towards tip. These characteristic material properties, in addition to the geometry of both stingers, provide the necessary stiffness which prevents structural failure during penetration. The region near the tip has the minimum elastic modulus which is higher on the order of average elastic modulus of human skin^10^. In addition, the tips of wasp and honeybee stingers are very sharp with required stiffness and also have a refined insertion mechanism which enables them to puncture and penetrate easily through the human skin.

**Figure 5:**
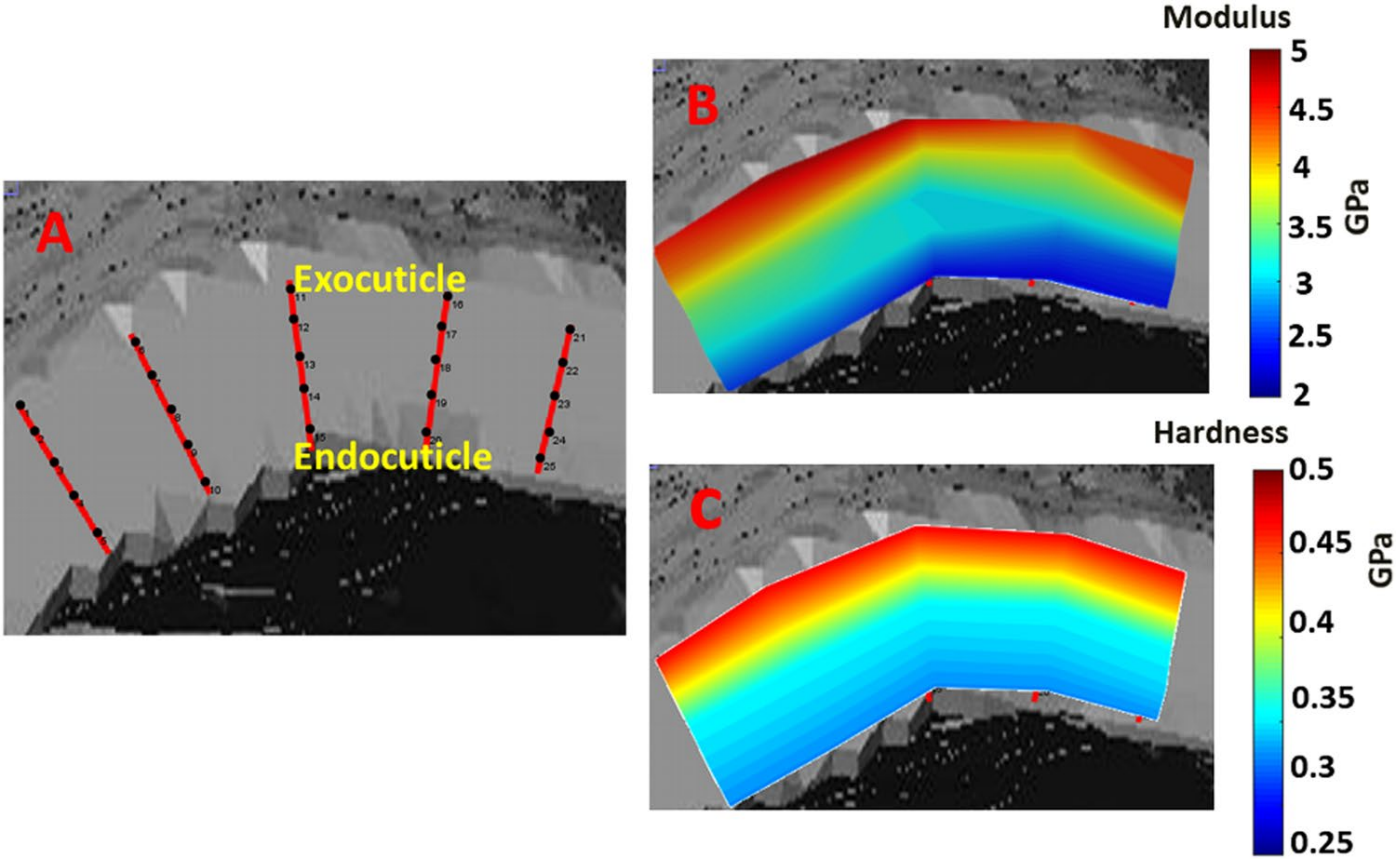
Quasi-static nanoindentation results at a cross section of the wasp stinger, (**a**) The approximate region at basal cross section where nanoindentation was performed, (**b**) Approximate nanoindentation sites, (**c**) Elastic modulus mapping, and (**d**) Hardness mapping. The cross section of the wasp stinger has hierarchical laminated structure and the mechanical properties are reduced from exocuticle to endocuticle.

**Figure 6:**
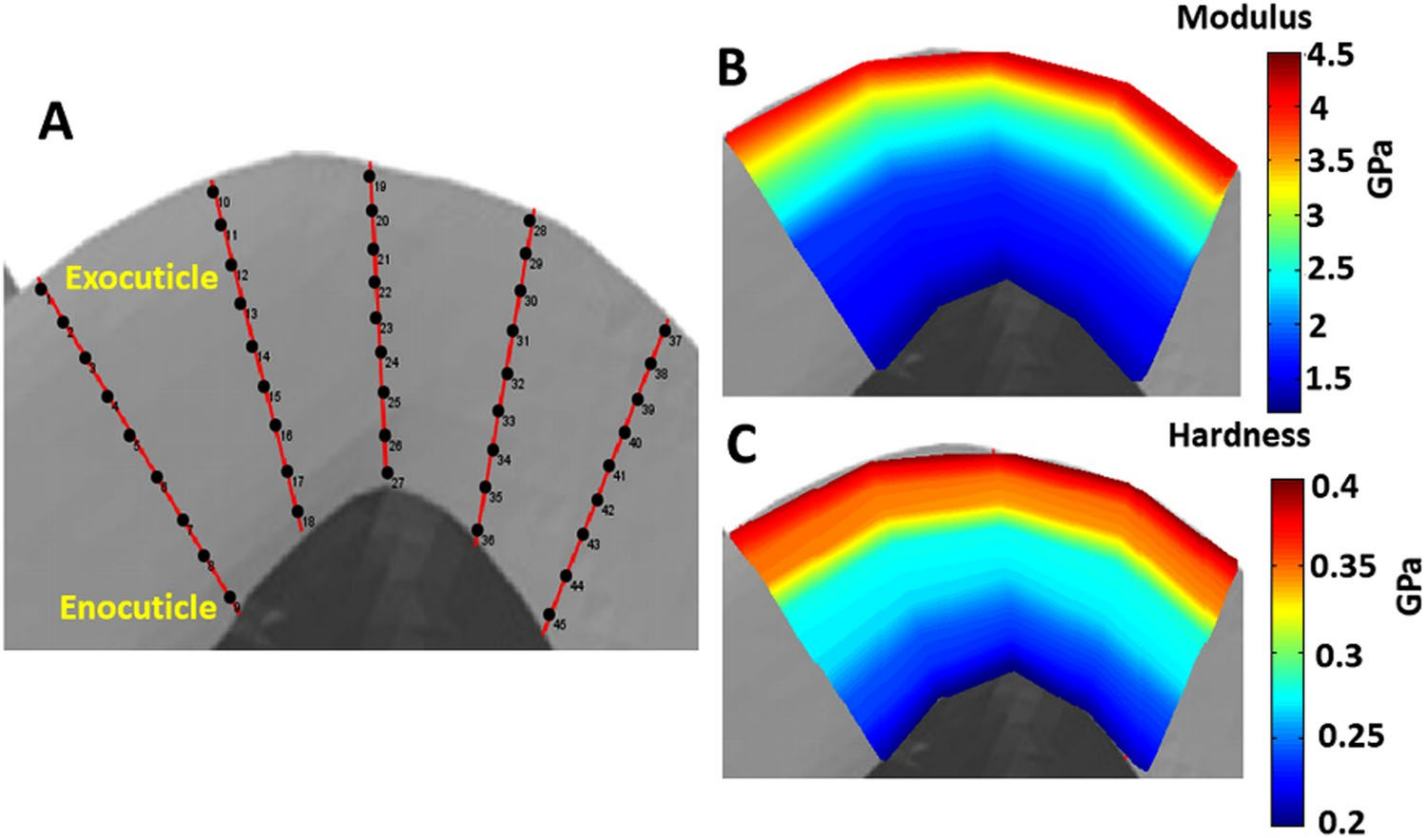
Quasi-static nanoindentation results at cross section of honeybee stinger, (**a**) The approximate region at basal cross section where nanoindentation was performed, (**b)** Approximate nanoindentation sites, (**c**) Elastic modulus mapping, and (**d**) Hardness mapping. The mechanical properties variation at the cross section of the honeybee stinger has the same trend as wasp stingers.

A quasi-static nanoindentation was performed at the stylet cross section of the wasp and honeybee stingers. The nanoindentation sites and elastic modulus and hardness maps for wasp and honeybee stingers are shown in Figs 5 and 6, respectively. From the elastic modulus and hardness mapping, it is clear that both the elastic modulus and hardness have a negative gradient along the cross sectional length from exocuticle to endocuticle. These moduli and hardness distributions strongly favor the laminated structure of the cuticles^11^ which is expected to imply hierarchy in the microstructure of the stingers.

The quasistatic nanomechanical analysis on the dorsal surface of the stingers i.e. on the exocuticle was performed. The negative gradient of mechanical properties along the longitudinal direction from base to tip was observed along with the negative gradient in the cross section from exocuticle to endocuticle. For spider fang^8,12^ and scorpion stinger^2^ and other biological structures^13^, the gradient architecture and mechanical properties provide more structural stiffness and fracture toughness. Most biological structures having the ability to penetrate through a hard substrate (like spider fang, scorpion stinger, parasitoid ovipositor^14^ etc.) have a tip with higher stiffness. But, in our study of mosquito labrum^15^, the nanomechanical properties revealed that the tip of the labrum has the least amount of hardness. It is believed that because of the soft and compliant tip, lesser pain is experienced during mosquito piercing.

To summarize, the stingers of wasps and honeybees are mechanically and structurally graded. The geometry of the stingers changes along its longitudinal direction as observed by structural analysis. The mechanical properties also decrease along the longitudinal direction of the stingers from base to tip, and along the cross sectional layers from exocuticle to endocuticle.

### Mechanical analysis through numerical modelling

**Figure 7:**
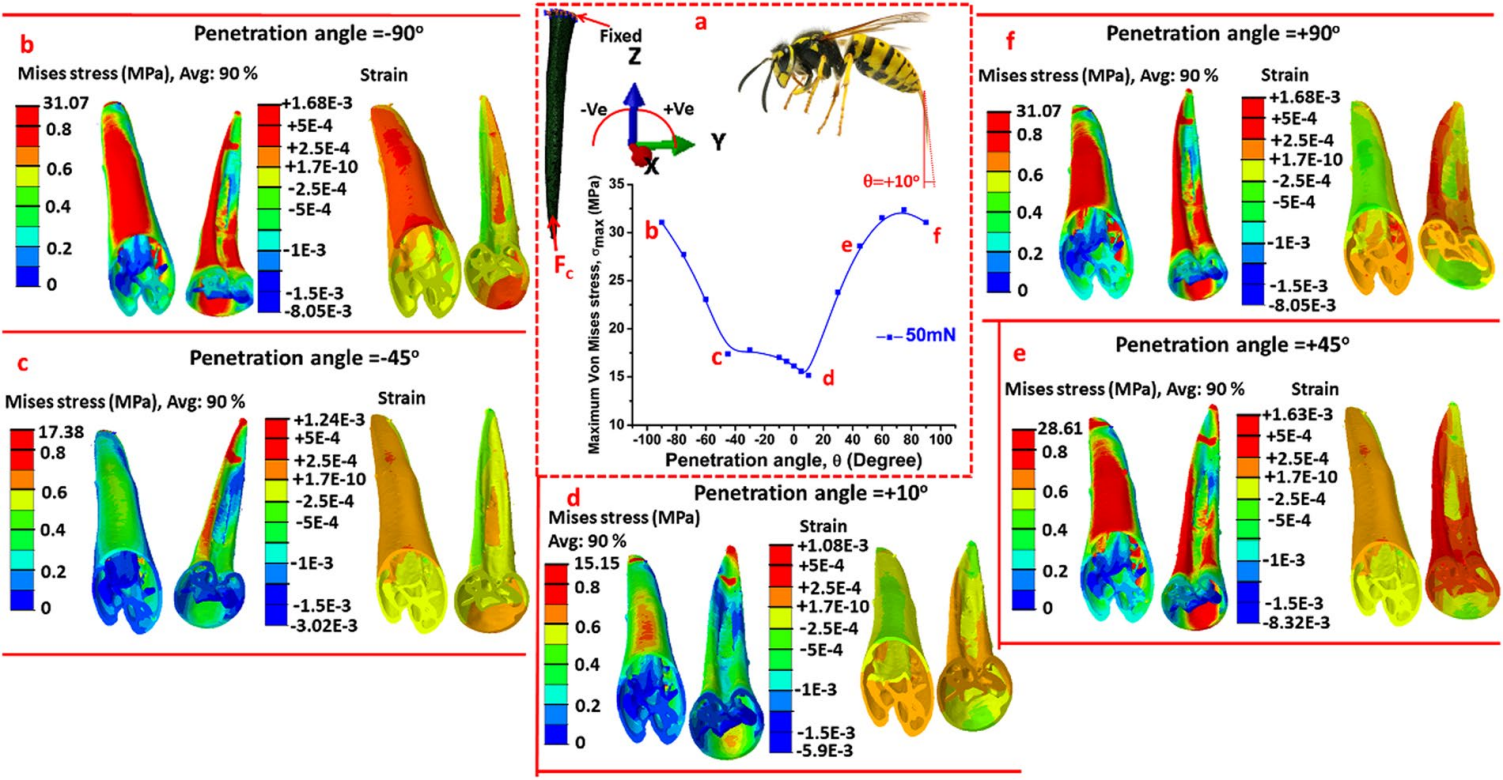
(**a**) Variation of maximum von Mises stress with the angle of applied concentric compressive force (F_c_) under cantilevered condition of the wasp stinger, (**b**) Stress and strain distribution at angle −90°, (**c**) Stress and strain distribution at an angle −45°, (**d**) Stress and strain distribution at an angle 10°, (**e**) Stress and strain distribution at a penetration angle +45°, and (**f**) Stress and strain distribution at +90°. The maximum von Mises stress (σ_max_) has a minimum value at the penetration angle of about +10°. As compared with other penetration angles, the stinger experiences the minimum stress and strain at this penetration angle, and the wasp finally adopts this angle to insert its stinger into objects.

The boundary conditions and all output results for the numerical simulation for wasp stingers are shown in Fig. 7. As shown in Fig. 7a, the base of the stinger was made planar in the X-Y plane, and the concentric force, F_c_ was applied at the tip of the stinger along the Z direction. The angle of applied force was denoted by θ which was varied from −90° to +90°, keeping the magnitude constant. For convention, the applied angle of force is treated as penetration angle.

In Fig. 7, the applied concentric force of the wasp stinger was 50 mN, and the von Mises stress distribution and strain distribution maps for specific penetration angles are presented. In Fig. 7a, 90% of the maximum von Mises stress, (σ_max_), as a function of penetration angle is plotted. The σ_max_ is very sensitive with the penetration angle. At a penetration angle of 10°, σ_max_ is minimum whereas at +90° and −90° penetration angle, it is maximum. The wasp gradually regulates its penetration angle from +18° to +9° during the penetration process^1^. In the early stage of penetration, the wasp applies force on the object surface by pressing the stinger tip and also by rotating the stinger. Gradually, it increases the pressing force, and in parallel, it adjusts the rotating angle. When the pressing force reaches an optimum level, the puncture occurs. At this stage, the rotating angle becomes almost fixed and the wasp deeply inserts its stinger. For this condition, the stinger can be treated as fixed at its base and its tip is subjected to a concentric compressive force. Indeed, this fixed angle significantly contributes to effective penetration of the stinger with minimum effort.

It is observed in the σ_max_ versus θ plot, the σ_max_ value is in a lower range at approximately 10°. The stress and strain distributions at different specific penetration angles are presented in Fig. 7b–f, respectively. The stress distribution for both the dorsal and ventral side of the stinger at the penetration angle of 10° is shown in Fig. 7d. The small region near the tip and in the subapical region experiences very high stresses. As the concentric force is applied at the tip, the subapical region always undergoes high stress in all represented penetration angles. The σ_max_ at 10° is obtained as σ_D_ = 15.15 MPa. The other sections of the stingers are very low stressed regions having stress range 0 to 0.3 MPa. At other penetration angles, the stress distributions as represented in Fig. 7b,c,e,f, show that high stress regions develop all over the dorsal surface and ventral surface and also at their interface.

The strain distribution at these specific penetration angles is also presented in the Fig. 7. At the penetration angle of +10°, the dorsal surface at the basal region undergoes compressive strain of very small magnitude (≈−2.5 × 10^−4^ to −5 × 10^−4^). Whereas, the dorsal surface at the apical region undergoes small tensile strain of ≈+2.5 × 10^−4^ under the effect of the applied compressive concentric force. The ventral surface also undergoes very small tensile strain, on the order of about +2.5 × 10^−10^. If the strain distribution at the other penetration angles is investigated, the magnitude of either the tensile or the compressive strain becomes higher. At the penetration angle of +10°, the σ_max_ is minimum and the overall strain is also minimum. Thus, the wasp adopts the penetration angle near the vicinity of +10°. When the magnitude of the applied force was also varied, it was observed that the force amplitude did not affect the angle at which the stress becomes minimum.

**Figure 8:**
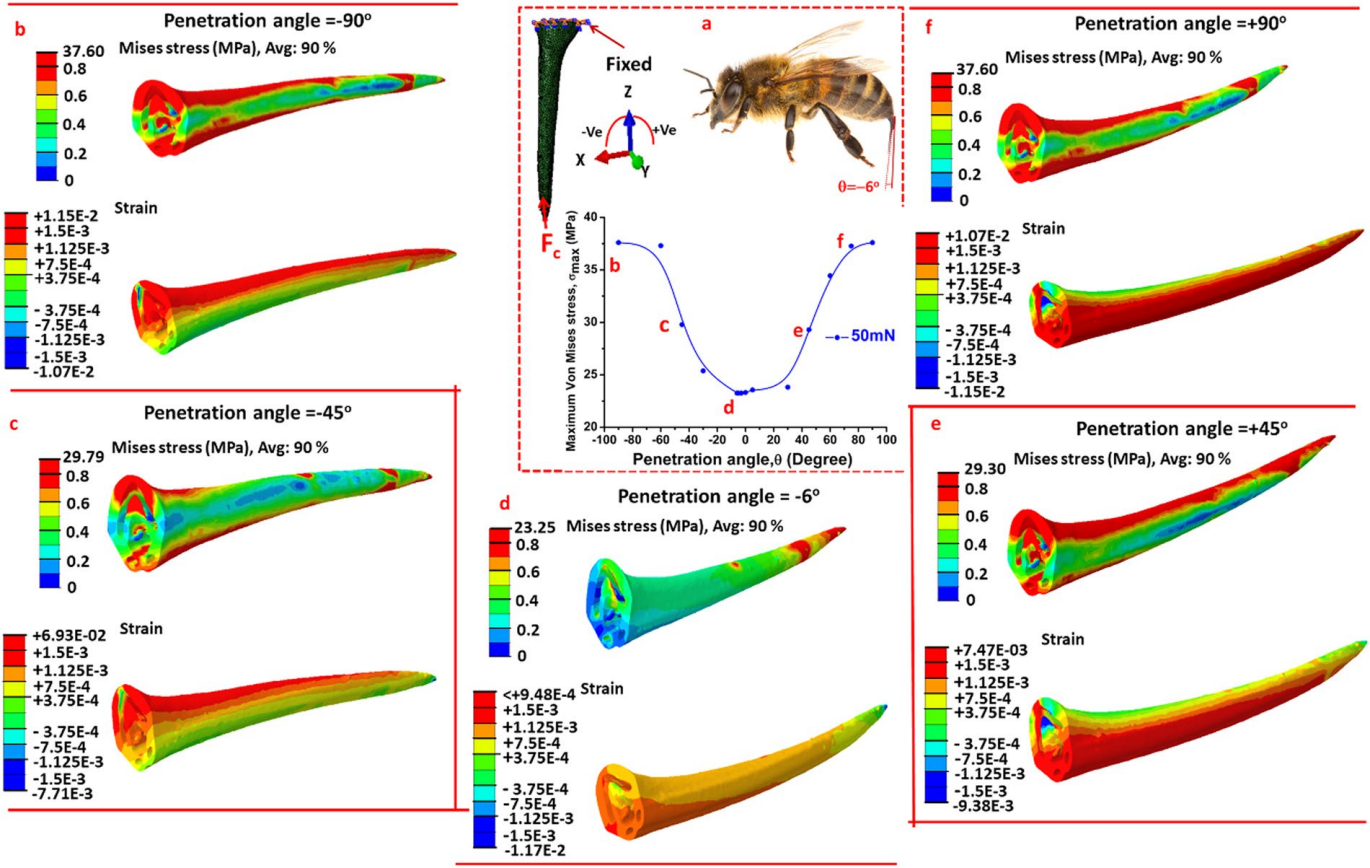
(**a**) Variation of maximum von Mises stress with the angle of applied concentric compressive force (F_c_) under cantilevered condition of honeybee stinger, (**b**) Stress and strain distribution at angle −90°, (**c**) Stress and strain distribution at angle −45°, (**d**) Stress and strain distribution at angle +10°, (**e**) Stress and strain distribution at penetration angle +45°, and (**f**) Stress and strain distribution at +90°. Honeybee finally adopts the angle of about −6° to insert its stinger into objects as the maximum von Mises stress and strain become minimum in that angle.

The boundary conditions and all output results for the numerical simulation for honeybee stingers are shown in Fig. 8. The stress and strain distributions of the honeybee stinger at different penetration angles are exhibited in Fig. 8b–f, respectively. In the honeybee stinger, the identical boundary conditions and procedures as used in the wasp stinger were applied to obtain its mechanical behavior. In Fig. 8a, the boundary condition along with the variation of the 90% of the maximum von Mises stress, (σ_max_) with the penetration angle, θ are presented. The σ_max_ at the penetration angle of θ = −6° is minimum. In strain distribution, the magnitude of either the tensile or the compressive strain becomes higher at other penetration angles in comparison with θ = −6°. Thus, the honeybee inherently takes the penetration angle to nearly −6° for effective insertion of its stinger into the skin. Like the wasp stinger, the honeybee also regulates its penetration angle from −6° to −13° at the different stages of penetration^1^ and follows the similar mechanism. The only difference is that the reverse facing barbs of the honeybee stinger allow the honeybee stinger to puncture the human skin or any object by a lesser amount of insertion force^3,16^. The average penetration angle of the honeybee stinger is reported as −8.3° ^10^. The penetration force of the honeybee stinger into the rabbit skin is about 5.8 mN^16^ and into porcine skin is about 2–3 mN^9^. But the pullout force from the rabbit skin is very high about at 113 mN. The interlocking of tissue fibers under barbs leads to an increase the adhesion of the stinger which results an enhancement of pull out force. This stress concentration at the sharp tip which can be considered as a contact point during its penetration helps the stinger to puncture the skin^16^. Like the wasp stinger, the minimum stress occurs at a fixed angle irrespective of applied force amplitudes.

**Figure 9:**
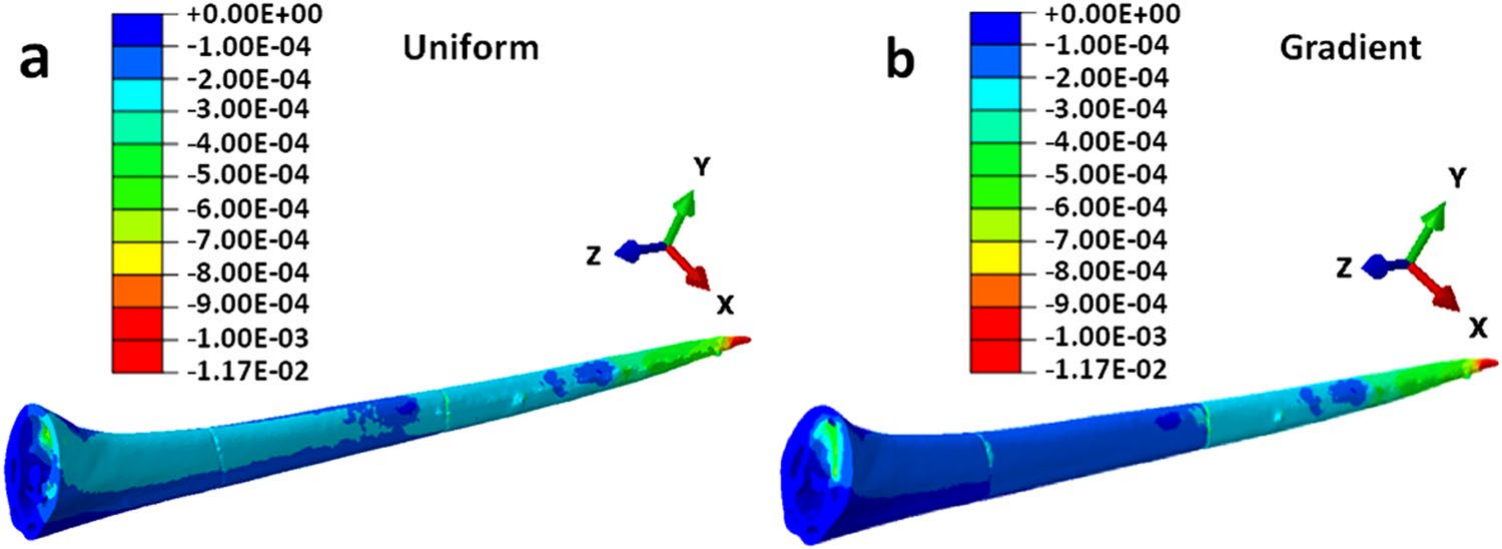
The compressive strain (E33) distribution pattern of the honeybee stinger; (**a**) stinger with uniform mechanical properties, and (**b**) stinger with gradient mechanical properties. For uniform stinger, relatively higher compressive strain spreads all over the stinger, whereas for gradient stinger it does not, and this justifies more mechanical stability of gradient stinger.

As discussed earlier, the gradient in structural and mechanical properties and hierarchical microstructure together enhances the mechanical stability of the biological structures which subsequently improves their survival in nature. To investigate the advantage of gradient mechanical properties on the mechanical behavior of the honeybee stinger during penetration, the strain distribution of the stinger in both cases, under the action 50 mN concentric force at its tip, is presented in Fig. 9. The strain as denoted by E33 is along the Z direction i.e. the axial direction (longitudinal) of the stinger. No difference in stress distribution has been observed in either case as the applied force and geometry were kept constant. Thus the stress distribution is not presented here. In the stinger having gradient mechanical properties, the non-deformed region near the base is higher. Thus the overall stiffness of the structure increases in gradient properties. More significantly, the tip has the lowest modulus in comparison to the other sections (basal and medial). Moreover, it has been also observed that the penetration angle is not influenced by the gradient mechanical properties.

To summarize, the wasp adopts a penetration angle of about +10° and the honeybee adopts it at about −6° to insert their stingers into objects. These angles have significant relevance in maintaining the structural stability of the stingers.

### Bioinspired design of micro syringe-needle

The detailed structural analysis, biomechanical evaluation and the role of insertion angle of wasp and honeybee stingers may significantly contribute to the design of a painless and minimally invasive micro syringe-needle for transdermal drug delivery, as well as to the design of other biomedical devices. From the current studies, the obtained gradient geometry and mechanical properties, the role of insertion angle and the sliding mechanism of paired lancets with the venom pumping mechanism^9,17^ can be mimicked to design a micro syringe-needle device.

**Figure 10:**
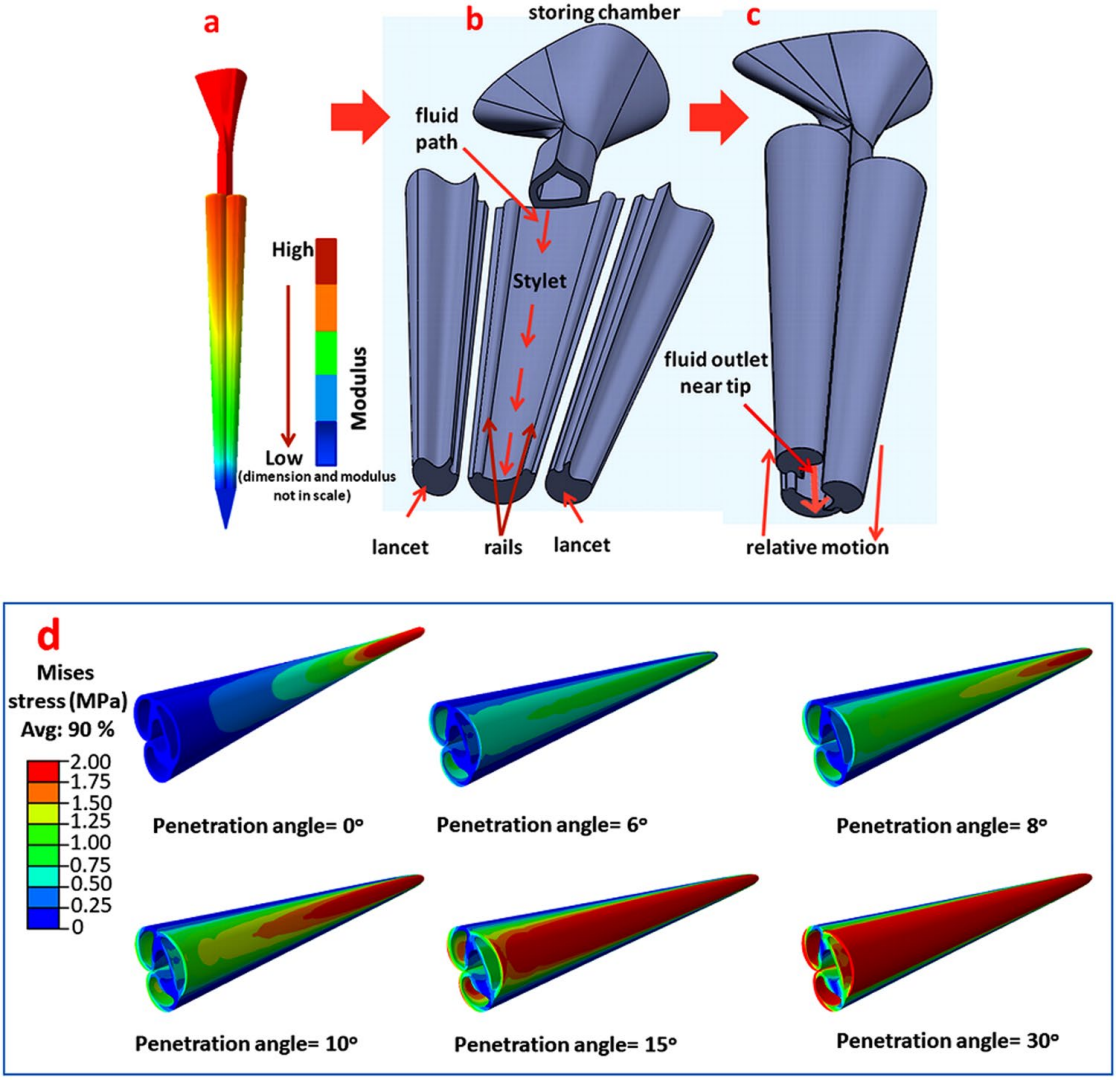
(**a**) The proposed design of an artificial microneedle mimicking the stingers, (**b**) The magnified view of the internal structure of the microneedle with all components (tip has been removed), (**c**) The relative sliding motion of lancets to generate an outlet near tip in magnified view, (**d**) The von Mises stress distribution in the microneedle at different penetration angles. The colour variation represents the functionally graded materials in the micro syringe-needle where the mechanical properties increase along the longitudinal direction from tip to base. A gap is generated near the tip due to sliding relative motion of lancets on the rails of the stylet, and the numbing fluid stored in the storing chamber will be pumped through the one-way valve from the chamber to the targeted body. In (**d**), the minimum stress distribution has been observed at the specific penetration angle of 6°.

The proposed design of a micro syringe-needle is schematically shown in Fig. 10. Like wasp and honeybee stingers, the microneedle should have decreasing diameter from base to the tip and should be made of materials that have gradient mechanical properties with a minimum at the tip and maximum at the base. The advantage of the structures having gradient geometry and mechanical properties, has been already described. Because of the higher overall stiffness coupled with a low stiff tip, this micro-needle is expected to be effective in obtaining less painful insertion with minimal dermal injuries, while maintaining mechanical durability. In addition, a concept of penetration angle can be implemented into the insertion mechanism of the microneedle. If a micro-needle with a geometry that is similar to stingers (primarily the curvature at the medial region can be adopted), insertion in a particular angle will give minimum stress (as shown in Fig. 10d), which should improve the mechanical durability and performance of the micro-needle. In the simulation of the micro-needle, the methodology applied in the stinger has been repeated. Although, a number of researchers have proposed the bio-inspired designs of micro-needles for biomedical applications^17–20^, the design features of the proposed prototype have never been investigated. It is predicted that the customized design of this micro-needle will have the capability of insertion and pull out by a comparatively low stiff tip using a moderate puncturing and pull out force. Different biomedical devices like hard tissue biopsy needles, surgical sutures, etc. may also be designed using this concept of painless insertion and removal, minimal insertion force and tissue adhesion without any damage.

In addition, the sliding mechanism, as observed in stingers^1,9^, may be implemented in the functioning of micro syringe-needle system for delivery of a numbing fluid to minimize pain. Because of this sliding motion, a gap for numbing fluid transportation will be generated near the tip. The fluid will be stored in the storing chamber of the microneedle, which will be transported through the one-way valve into the storing chamber, designated fluid propagation path and the gap near the tip. The source for the generation of force to purge the fluid into the targeted body is not clear yet. It may be generated by squeezing the storing chamber. Alternatively, a mechanism to generate negative pressure may be developed by the sliding motion at paired lancet. This negative pressure can drive the fluid into the targeted body. The functioning of this syringe-needle system is unique and may be conceptualized and incorporated into the design of a new syringe-needle system.

To summarize, a conceptual schematic of a medical micro syringe-needle system is presented with a capability for painless insertion and extraction, minimal dermal injuries and that is mechanically durable and has suitable biocompatibility.

## Conclusions

In this study, the structure, nanomechanical properties and functionalities of wasp and honeybee stingers have been investigated through experimentation and numerical modelling. The 3D micro-computed tomography reveals the structural features. The quasi-static nanoindentation instrument evaluates the biomechanical properties of wasp and honeybee stingers. Both wasp and honeybee stingers have structures that are simple but have mechanical functionalities which lead to refined insertion and venom propagation into the victim’s body.

The structure of the honeybee stinger slightly differs from the wasp stinger. The honeybee stinger has straight geometry, with reverse facing barbs in the stylet whereas, the wasp stinger has curved geometry with the presence of a reinforcement rib at its stylet. The quasi-static nanoindentation measurement establishes the presence of a negative gradient of elastic modulus and hardness from base to tip along the longitudinal direction of the both wasp and honeybee stingers. The quasi-static nanoindentation results along the basal cross section of both wasp and honeybee stingers implies that the stinger cuticle is made up of laminated microstructure.

Similar structural gradients i.e. the decreasing cross section from base to tip are present in spider fangs and scorpion stingers. However they have more intrinsic curvature and have an opposite mechanical property gradient along the longitudinal direction from base towards tip.

The numerical simulation of both the wasp and honeybee stingers, utilizing µ-CT images and measured mechanical properties from nanoindentation is used to study the mechanical behavior of stingers during insertion. The stress and strain distribution of wasp and honeybee stingers are at an optimum level at +10° and −6°, respectively, which are the angles of applied constant force to the tip of the cantilevered stingers during simulation. Thus, the wasp and honeybee stingers adopt these angles for effective penetration of their stingers into the victim’s body. The insights of structural features, gradient geometry, functionally gradient mechanical properties and the mechanical performance of the stingers during insertion can be used for design of a sophisticated bioinspired micro syringe-needle or other devices in biomedical engineering applications.
